# Palladium-catalyzed dual C–H or N–H functionalization of unfunctionalized indole derivatives with alkenes and arenes

**DOI:** 10.3762/bjoc.8.198

**Published:** 2012-10-11

**Authors:** Gianluigi Broggini, Egle M Beccalli, Andrea Fasana, Silvia Gazzola

**Affiliations:** 1 Dipartimento di Scienza e Alta Tecnologia, Università dell’Insubria, Via Valleggio 11, 22100 Como, Italy; 2 DISFARM, Sezione di Chimica Generale e Organica “A. Marchesini” Università di Milano Via Venezian 21, 20133 Milano, Italy

**Keywords:** alkenylation, arylation, C–H functionalization, indoles, N–H functionalization, Pd catalysis

## Abstract

This review highlights the development of palladium-catalyzed C–H and N–H functionalization reactions involving indole derivatives. These procedures require unactivated starting materials and are respectful of the basic principle of sustainable chemistry tied to atom economy.

## Introduction

The development of mild and selective reactions for the direct conversion of carbon–hydrogen bonds into carbon–carbon and carbon–heteroatom bonds is a challenging goal in organic chemistry [1–3]. The coupling of C–H/C–H or C–H/N–H bonds in an oxidative system is an attractive target since hydrogen or water would be the only formal byproduct. In this field, (hetero)aryl–(hetero)aryl, (hetero)aryl–alkenyl, and (hetero)aryl–alkyl reactions represent some of the most important tools for planning the synthesis of a wide range of different kinds of molecules. Synthetic approaches using unfunctionalized reagents rather than halogenated compounds have attracted strong attention, above all due to their atom- and step-economical characteristics.

Thus, the applicability of these transformations on the multiscale level paves the way to cheaper processes, resulting in minimal waste production and raising the possibility of application in multistep synthetic sequences. Many transition metals, including Pd, Au, Ru, Rh, Cu, and Pt, have been proven to be highly efficient for the formation of new bonds without prefunctionalized starting materials [4–10]. Among the transition metals suitable for this purpose, palladium plays a pivotal role due to its versatility in different synthetic protocols and tolerance towards many functional groups, often avoiding the need for protecting-group chemistry [11–16]. Moreover, palladium-catalyzed reactions involving ethylenic double bonds can also lead to domino processes such as carboaminations [17–19], diaminations [20–21], aminooxygenations [22–23], and aminohalogenations [24–25]. The most common reactions of C–H functionalization on unactivated substrates typically occur with electrophilic Pd(II) catalysts and require an oxidizing agent in order to make possible the reoxidation of the Pd(0) species, generated in the final elimination step, for a new catalytic cycle [12–15].

The well-established features of natural or man-made compounds containing an indole backbone are of wide interest in pharmacological and agrochemical fields [26–28]. Thus, indole and carbazole nuclei are used in medicine for their antibacterial, antimicrobial, and anti-inflammatory effects and occupy a relevant role in the discovery of active antitumor drugs [29–31]. Carbazole derivatives also find applications in organic materials as chromophores and photoconductors [32]. For several years, the development of methodologies concerning indole synthesis and functionalization has been one of the most attractive goals in organic chemistry [33–39]. In the search for clean and sustainable synthetic protocols suitable to construct and convert the indole core motif into more complex structures, palladium-based catalytic systems were proven to be fruitful tools for organic chemists [40–42].

This review highlights methodologies based on the use of palladium catalysts, devoted to the functionalization of indole derivatives involving carbon–hydrogen and nitrogen–hydrogen bonds. The synthetic procedures are classified as intermolecular and intramolecular alkenylations, arylations, and domino processes.

## Review

### Intermolecular reactions involving alkenes

**Scheme 1:**
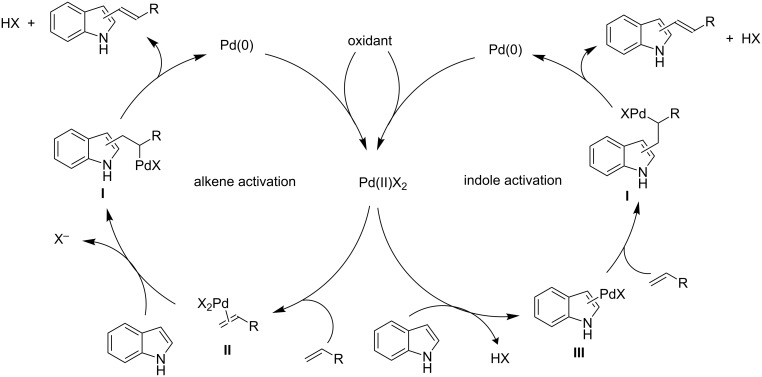
Typical catalytic cycle for Pd(II)-catalyzed alkenylation of indoles.

Alkenylation reactions of indoles run through a key C–H activation step involving an electrophilic palladation and an electron-deficient Pd(II) catalyst. The mechanism of these reactions involves the generation of a σ-alkyl complex **I**, which is the rate-determining step of the reaction, and conversion into the alkenylindole by a *syn*-β-hydride elimination process (Scheme 1) [43–45]. Beside the formation of the final product, the last step results in the elimination of HX and Pd(0) species, justifying the need for an oxidant agent to regenerate a Pd(II) species as active catalyst. Although seldom unambiguously determined, two alternative pathways, based either on “alkene activation” or “indole activation”, have been proposed to explain the formation of the σ-alkyl complex **I**. The former involves the coordination of the Pd(II) catalyst to the olefin, giving the π-olefin complex **II**, which is converted by nucleophilic attack of the indole into the intermediate **I**. On the other hand, an electrophilic attack of the Pd(II) catalyst on the indole to generate the indolyl-palladium(II) complex **III**, in turn susceptible to attack by the olefin, may be hypothesized as a plausible way to form the σ-alkyl complex **I**. In both pathways, indole may be involved directly at the C-2 or C-3 positions as well as preferentially at the C-3 position, in the latter case affording the final 2-substituted product by the intrinsic tendency toward C-3/C-2 rearrangement that is operative during the alkylation of indoles [46].

**Scheme 2:**
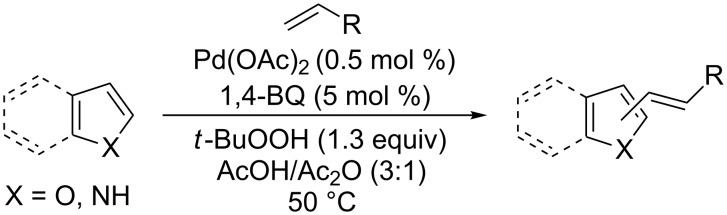
Application of Fujiwara’s reaction to electron-rich heterocycles.

In 1969, Fujiwara and Moritani reported the alkenylation of arenes catalyzed by Pd(OAc)_2_, using Cu(OAc)_2_ or AgOAc as oxidants [47]. This strategy provides a convenient method for the synthesis of olefins linked to heteroarenes, including indole, furan, and benzofuran rings (Scheme 2) [48]. Working with indole and methyl acrylates in the presence of Pd(OAc)_2_ and 1,4-benzoquinone in catalytic quantity with *tert*-butyl hydroperoxide as oxidant, 3-alkenyl-substituted products were obtained.

**Scheme 3:**
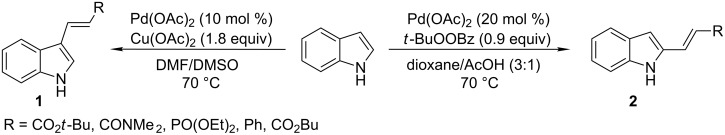
Regioselective alkenylation of the unprotected indole.

The synthetic value of the direct catalytic C–H alkenylation of the C-2 and C-3 positions of the unprotected indole nucleus was recognized under different conditions published several years ago. In 2005, Gaunt and co-workers disclosed a general method for the selective intermolecular alkenylation of the unprotected indoles through an oxidative palladium-catalyzed reaction (Scheme 3) [49]. The reaction can involve the formation of carbon–carbon or carbon–nitrogen bonds, which is strongly dependent on the solvent used. When the reaction is carried out in aprotic polar solvents, such as DMSO and DMF, with Cu(OAc)_2_ as reoxidizing agent, the alkenylation occurs at the 3-indolyl position, yielding products **1**. Conversely, the use of dioxane with the addition of acetic acid as a polar coordinating co-solvent in the presence of *tert*-butyl benzoyl peroxide, directs the selectivity in favor of the C-2 substituted indoles **2**. It should be noted that the same chemistry has been successfully extended to the pyrrole ring [50].

**Scheme 4:**
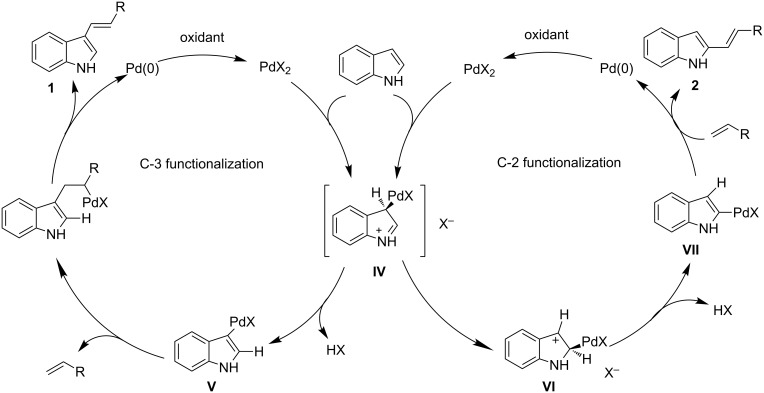
Plausible mechanism of the selective indole alkenylation, adapted from [49].

A rational explanation for the outcome of these reactions is described in Scheme 4. In both cases, intermediate **IV** is involved as the result of a direct palladation at the C-3 position. Working under neutral conditions, a proton can be easily removed from **IV** by the anion formed from the initial palladium salt with generation of the 3-indolyl-palladium complex **V**, which evolves a Heck-type reaction to give the 3-alkenylindoles **1**. Conversely, the deprotonation of the C-3 position is difficult in acidic medium, favoring the transfer of metal species to the 2-indolyl carbon of **IV**, activated as an iminium carbon. The so-formed intermediate **VI** undergoes loss of HX with generation of the complex **VII**, which finally reacts with alkenes giving the 2-alkenylindoles **2** and a Pd(0) species.

**Scheme 5:**
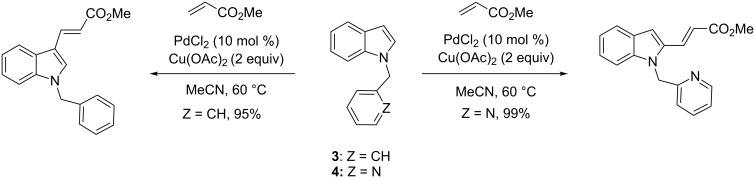
Directing-group control in intermolecular indole alkenylation.

An alternative approach to address the regioselective alkenylation of the C-2 position is based on the directing control of a group attached to the indole nitrogen. Under the same conditions, i.e., PdCl_2_ as catalyst and Cu(OAc)_2_ as oxidant in acetonitrile at 60 °C, alkenylation of *N*-benzyl-protected indole **3** took place selectively at the C-3 position, while the reaction of the *N*-(2-pyridylmethyl)-substituted indole **4** resulted in the functionalization of the C-2 position by directing coordination to the pyridyl nitrogen (Scheme 5) [51].

**Scheme 6:**
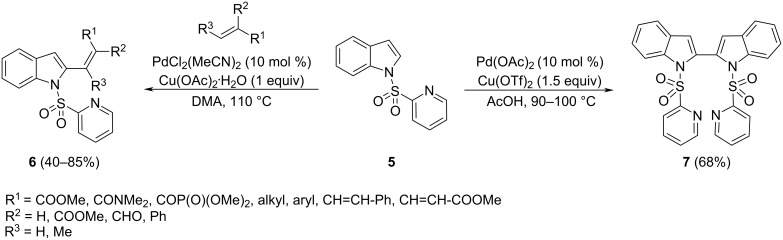
Direct C–H alkenylation of *N*-(2-pyridyl)sulfonylindole.

The control of the regioselectivity in the Pd(II)-catalyzed C–H alkenylations towards the indole C-2 position can be exerted by the *N*-(2-pyridyl)sulfonyl group, which can be easily installed and removed [52–53]. The reaction of **5** with a wide range of mono-, 1,1- and 1,2-disubstituted alkenes in the presence of a catalytic system based on PdCl_2_(MeCN)_2_ (10 mol %) and Cu(OAc)_2_ (1 equiv) in DMA, furnishes the product **6** in moderate to good yield (Scheme 6). A mechanism including an electrophilic palladation involving the pyridinyl chelation was thought to be plausible taking into account the outcome of the reaction performed on isotopically labeled substrates as well as by kinetic studies of variously substituted indoles. This *N*-(2-pyridyl)sulfonyl-directing strategy has also been extended to the development of a protocol for the intermolecular, dehydrogenative homocoupling of indole, providing 2,2’-bisindoles **7**.

**Scheme 7:**
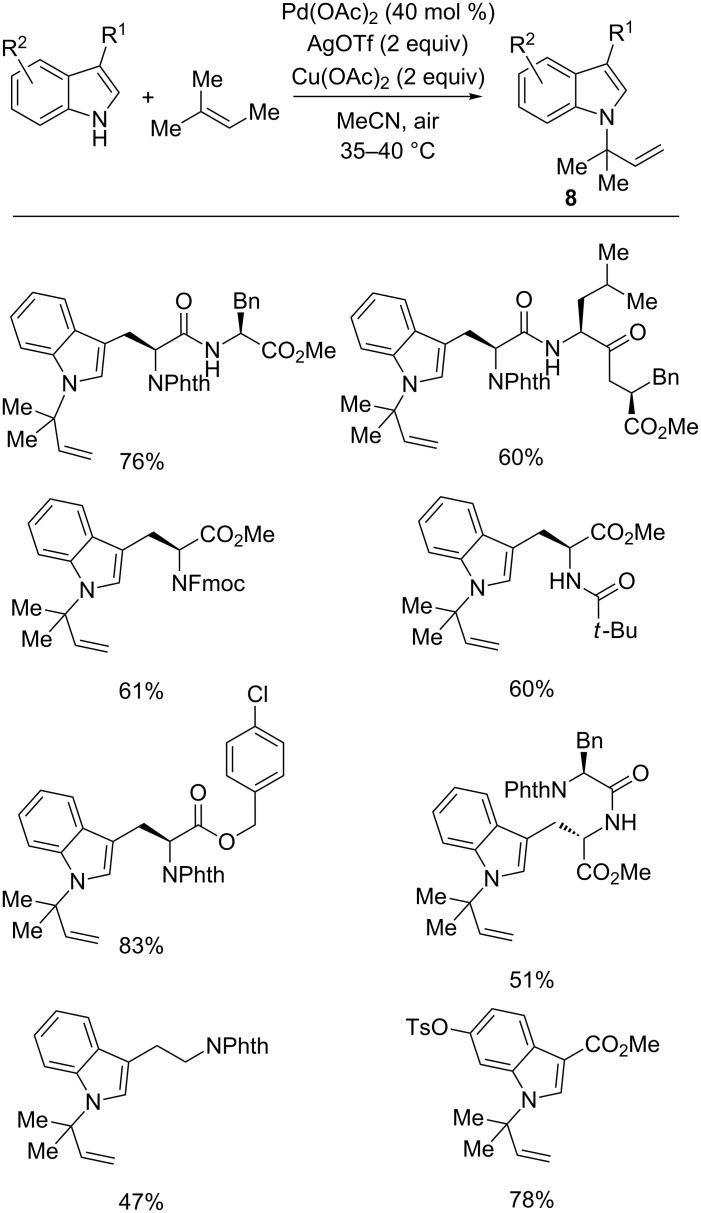
*N*-Prenylation of indoles with 2-methyl-2-butene.

Intermolecular Pd(II)-catalyzed N–H functionalization has also been successfully used to achieve *N*-substituted indoles. Coupling of indole and 2-methyl-2-butene in the presence of Pd(OAc)_2_ (40 mol %), Cu(OAc)_2_ and AgOTf as the co-oxidants in MeCN constitutes a simple route to *N*-prenylated indoles **8** (Scheme 7) [54]. This mild reaction, which exhibits broad functional-group tolerance, can be successfully performed for the prenylation of tryptophan and tryptamine derivatives, as well as peptides containing tryptophan.

**Scheme 8:**
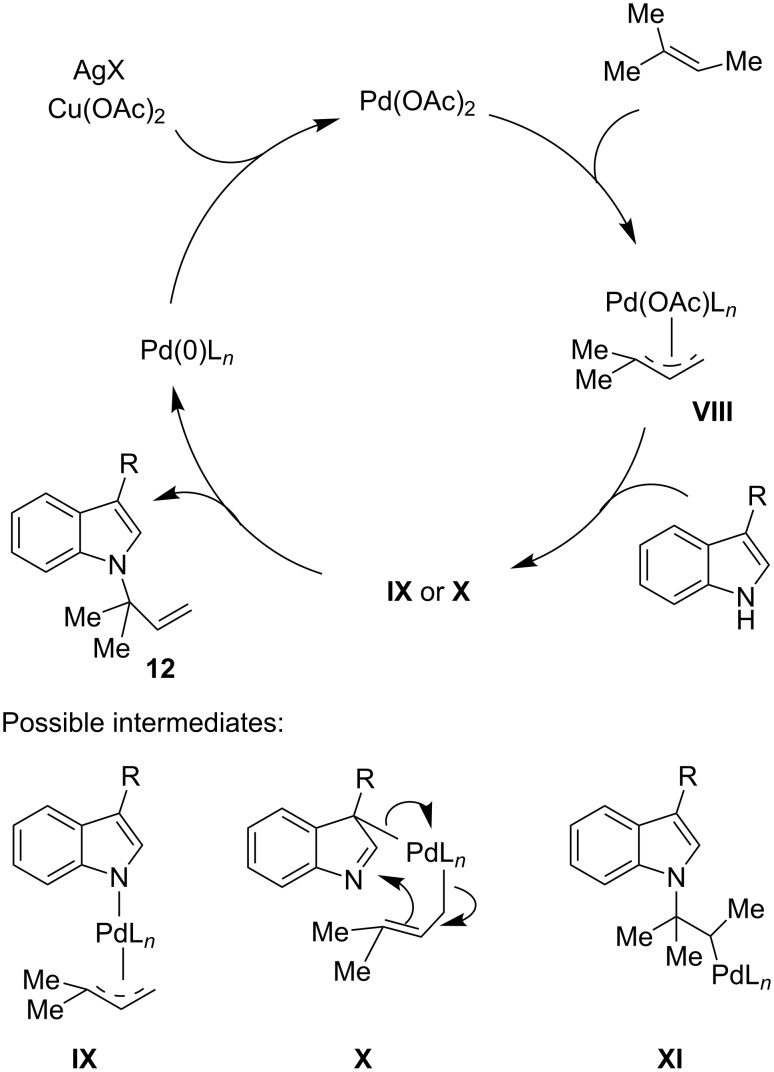
Proposed mechanism of the *N*-indolyl prenylation.

Taking into account some experimental evidence obtained from the use of 2-methyl or 2-deuterium-substituted indoles and from [1,1,1-D_3_]3-methyl-2-butene, the mechanism shown in Scheme 8 was thought to explain the outcome of the reaction. Firstly, Pd(II) catalyst promotes the formation of the π-allyl-palladium complex **VIII**, which can evolve by coordination of the N-1 or C-3 positions of the indole nucleus giving the palladium complexes **IX** and **X**, respectively. The latter quickly converts into the σ-alkyl-palladium intermediate **XI** by a Claisen-type rearrangement that involves the metal species. A mechanism through the typical π-olefin-palladium complex as the precursor of the σ-alkyl-palladium complex **XI** cannot, however, be ruled out. In every case, a Pd(0) species was released from **XI** and reoxidized with the Ag(I) and Cu(II) salts.

### Intermolecular reactions involving arenes

The formation of homo-coupling products is one of the most common drawbacks in intermolecular reactions between arenes without preactivation of the substrates. In 2006, Lu and co-workers reported one of the first articles providing conditions to access asymmetric biaryl compounds by dual C–H functionalization [55].

**Scheme 9:**
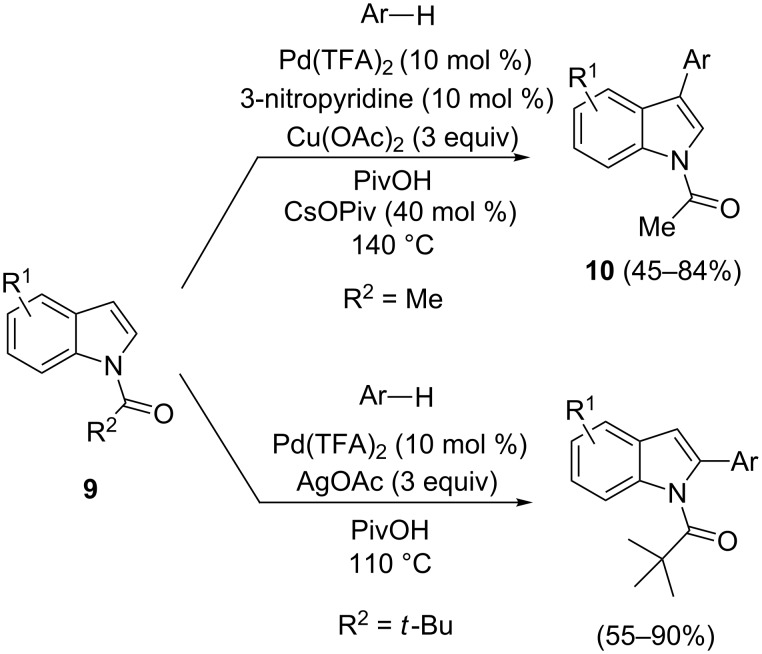
Regioselective arylation of indoles by dual C–H functionalization.

In 2007, Fagnou and co-workers combined, in a single catalytic cycle, the reactivity of electron-deficient palladium(II) complexes with electron-rich arenes (through an electrophilic C–H activation mechanism) and the reactivity of some Ar-Pd(II) complexes with arenes (through a proton-transfer palladation mechanism), depending on the C–H acidity rather than the arene nucleophilicity. Synthetic procedures based on this strategy allowed the direct arylation at C-2 and C-3 positions of indoles **9** with a high degree of regioselectivity (Scheme 9) [56–57]. 3-Arylindoles **10** were selectively achieved on *N*-acylindoles by using catalytic Pd(TFA)_2_ and a stoichiometric amount of Cu(OAc)_2_. The use of additives, such as 3-nitropyridine and caesium pivalate, was proven essential to achieve optimized conditions.

**Scheme 10:**
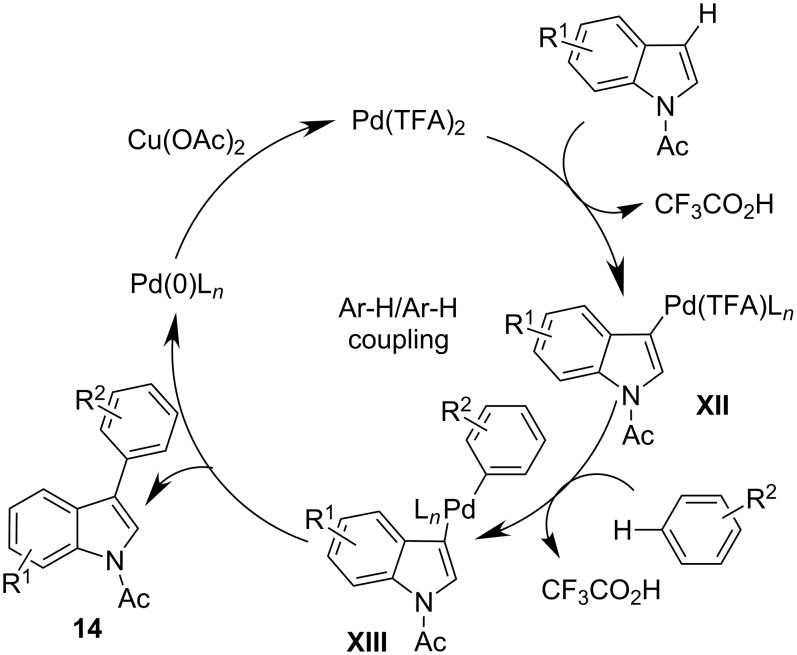
Plausible mechanism of the selective indole arylation.

It is plausible that the presence of pyridine can stabilize the final Pd(0) species favoring its reoxidation and avoiding the precipitation of palladium black. The use of AgOAc as oxidant induces an inversion of selectivity, improving the C-2 arylation process. A high level of C-2 selectivity was achieved by using the *N*-pivalyl-substituted indole in the absence of additives. From the mechanistic point of view, as depicted in Scheme 10, the C–H activation on the electron-rich indole, selectively directed by the strongly electrophilic behavior of the Pd(TFA)_2_ catalyst, is plausible giving the Pd(II) intermediate **XII**. The subsequent selective coordination of the arene generates the complex **XIII**, which in turn undergoes reductive elimination providing the final product and a Pd(0) species. The reoxidation of the latter giving the active Pd(II) catalyst completes the catalytic cycle.

In addition to the effect of Cu(OAc)_2_ and AgOAc as oxidant, a determinant role on the selectivity of direct C–H to C–H cross-coupling reactions was played by the acidity of the medium, as shown by reactions carried out in the presence of AcOH [58–59]. Based on experimental and computational data, a concerted metalation–deprotonation of the arene was hypothesized to explain the mechanism for C–H palladation.

### Intramolecular reactions involving alkenes

The first example of intramolecular indole alkenylation was reported in 1978 by Trost, who applied reaction conditions based on stoichiometric amounts of PdCl_2_(MeCN)_2_ and silver ions in the key step of the total synthesis of ibogamine alkaloids [60].

**Scheme 11:**
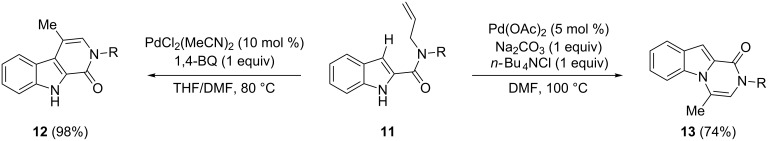
Chemoselective cyclization of *N*-allyl-1*H*-indole-2-carboxamide derivatives.

Palladium-catalyzed cyclization of *N*-allyl-1*H*-indole-2-carboxamides **11** is a fruitful procedure to access β-carbolinones **12** or pyrazino[1,2-*a*]indoles **13** (Scheme 11) [61–62]. The use of PdCl_2_(MeCN)_2_ as the catalyst with 1,4-benzoquinone as the oxidant in a mixture of DMF/THF resulted in the C-3 functionalization of the indole nucleus. Conversely, switching to Pd(OAc)_2_ with Na_2_CO_3_ as a base and Bu_4_NCl as an additive in DMF provided the indole N–H functionalization. This strategy has also been proven to be operative in effecting intramolecular alkenylation on a range of other electron-rich heterocycles, including pyrroles, furans and thiophenes [63–64].

**Scheme 12:**
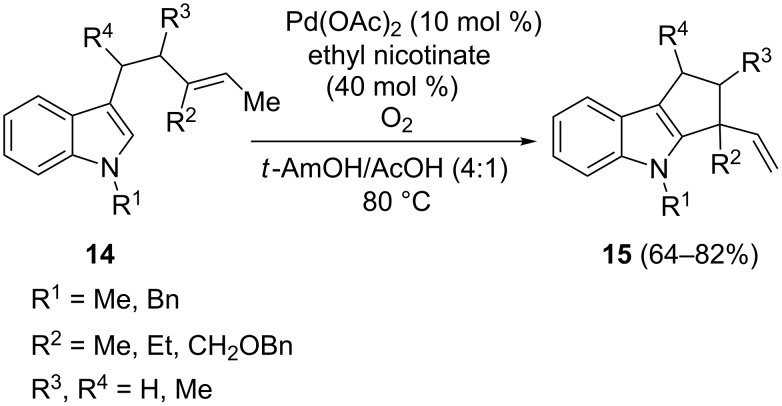
Intramolecular annulations of alkenylindoles.

The intramolecular Pd(II)-catalyzed reaction of the 3-alkenylindoles **14** gave rise to the carbocyclic 5-membered ring-fused products **15** (Scheme 12) [65–66]. This procedure involves O_2_ as the sole oxidant. Among the various pyridine ligands and solvents tested to optimize the conditions, 3-carbethoxypyridine in a polar solvent (i.e., *tert-*amyl alcohol/AcOH in 4:1 ratio) was proven to be the most effective in providing satisfactory yields. The oxidative cyclization led also to a new 6-membered ring, once again producing vinyl-substituted products. An analogous process for the direct intramolecular C–H functionalization of inactive alkenyl aryl ethers, giving benzofuran and dihydrobenzofuran derivatives, was successfully developed [67].

**Scheme 13:**
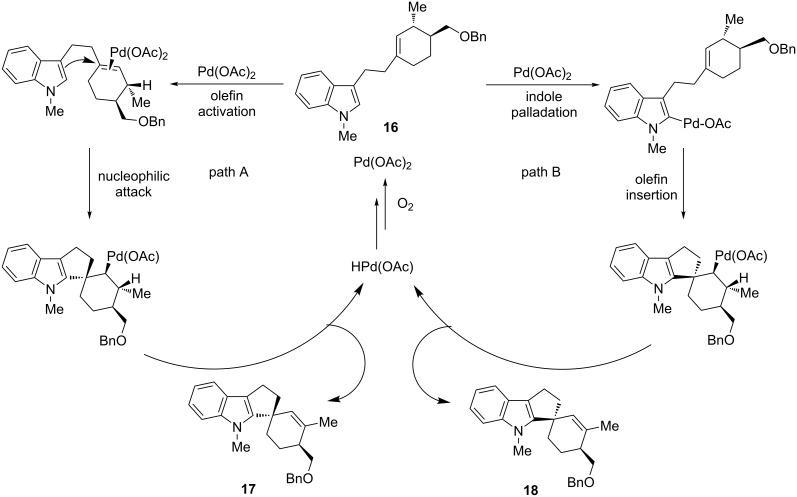
A mechanistic probe for intramolecular annulations of alkenylindoles, adapted from Ferreira et al. [66].

Both possible mechanistic pathways based on the initial coordination of the Pd(II) catalyst to the 2-indolyl position or to the carbon–carbon double bond, can be hypothesized for this reaction. Elucidation of the outcome of the reaction was achieved by cyclization of the diastereoisomerically pure cyclohexenylindole **16**, which could give the spiro-products **17** and **18** (Scheme 13). The sole formation of the annulated indole **18** as a single diastereoisomer suggests a mechanism that is strictly closer to the classical oxidative Heck reaction (pathway B) rather than to a Wacker-type reaction (pathway A). In fact, the formation of the product **18** is explainable by an indolyl palladation and a β-hydride elimination, which typically occurs in *syn* manner. The formation of the diastereoisomeric product **17** would have been justified by a nucleophilic attack of the indole on the π-olefin complex, which is known to occur in *anti* fashion, before the β-hydride elimination.

**Scheme 14:**
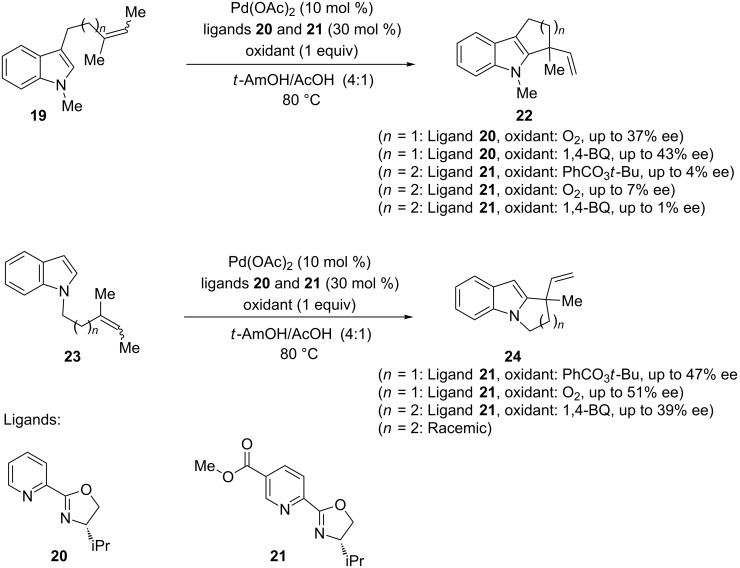
Asymmetric indole annulations catalyzed by chiral Pd(II) complexes.

Palladium–pyridine systems were subsequently investigated with chiral ligands to catalyze enantioselective processes involving alkenylindoles. Several enantioselective indole annulations with formation of a stereogenic quaternary carbon atom were performed by using chiral oxazoline ligands with pyridine or nicotine platforms (PyOx and NicOx, respectively) [68–69]. A moderate level of enantiocontrol (up to 51 % ee) was seen in 5-*exo-*trig cyclization of the 3-alkenylindole **19** (*n* = 1) in the presence of ligands **20** and **21**, to yield **22**, whilst the outcome of the 6-*exo-*trig cyclization of indole **19** (*n* = 2) resulted essentially in racemic products (Scheme 14). The same behavior, in terms of the degree of enantioselectivity depending on the ring size of the newly formed ring, was observed in the cyclization of the *N*-alkenylindole **23** to give the pyrrolo[1,2-*a*]indole **24** (up to 51% for 5-*exo*-trig cyclization).

**Scheme 15:**
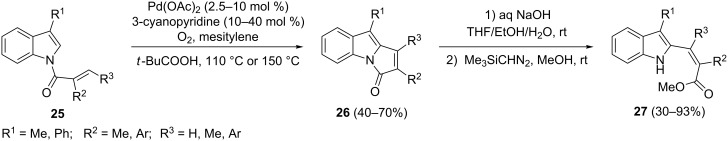
Aerobic Pd(II)-catalyzed *endo* cyclization and subsequent amide cleavage/ester formation.

A strategy involving an intramolecular C–H bond alkenylation of trisubstituted alkenes, followed by ring opening of the so-formed ring, was planned to achieve the diastereocontrolled formation of tetrasubstituted double bonds tethered to C-2 indole. The Pd(II)-catalyzed 5-*endo*-trig cyclization of *N*-alkenoylindoles **25** in the presence of 3-cyanopyridine as the ligand and under aerobic conditions afforded the tricyclic products **26** (Scheme 15) [70]. The subsequent amide cleavage carried out in aqueous NaOH and following ester formation by treatment with Me_3_SiCHN_2_ in methanol led to the 2-alkenylated indoles **27**.

**Scheme 16:**
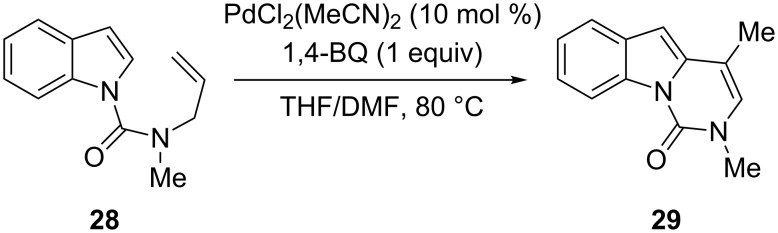
Synthesis of the pyrimido[3,4-*a*]indole skeleton by intramolecular C-2 alkenylation.

The pyrimido[3,4-*a*]indole skeleton **29** was proven to be accessible by intramolecular 6-*exo*-trig cyclization of the *N*-alkenylindole **28** with PdCl_2_(MeCN)_2_ as catalyst and 1,4-benzoquinone as oxidant in THF/DMF at 80 °C (Scheme 16) [51].

**Scheme 17:**
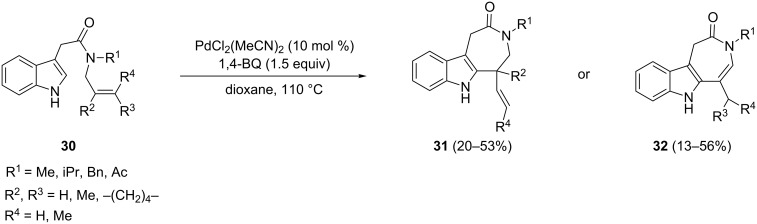
Synthesis of azepinoindoles by oxidative Heck cyclization.

Catalytic oxidative Heck reactions allowed also the construction of seven-membered ring-fused indoles. Readily available *N*-alkenyl-3(1*H*)-indoleacetic amides **30** were converted into the azepinoindole derivatives **31** or **32** by using the combination of PdCl_2_(MeCN)_2_, 1,4-benzoquinone and dioxane at 110 °C (Scheme 17) [71]. Although these reactions achieve only moderate yields, this strategy constitutes an alternative choice to the palladium-catalyzed cyclization of indole amides bearing a carbon–halogen bond to give medium and large ring-fused indoles [72].

Although a stoichiometric amount of Pd(OAc)_2_ is needed, intramolecular alkenylations of suitable 3-alkenylindoles in an atmosphere of molecular oxygen provided dihydroindoloazocine compounds that are key intermediates in the total synthesis of the austamide derivatives and the okaramine family of polycyclic bisindole alkaloids [73–74].

**Scheme 18:**
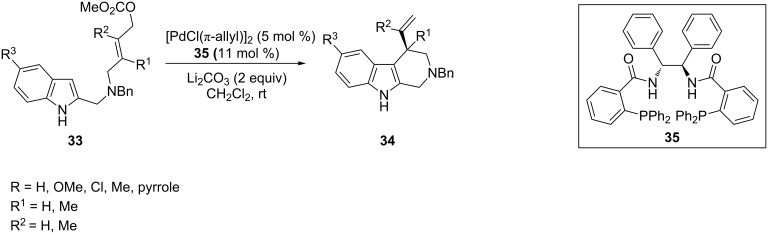
Enantioselective synthesis of 4-vinyl-substituted tetrahydro-β-carbolines.

Enantioselective synthesis of vinyl-substituted tetrahydro-β-carbolines and tetrahydro-γ-carbolines was performed starting from 2- and 3-alkenylindoles by Pd-catalyzed asymmetric allylic alkylation. A series of (*E*)-5-substituted indolylcarbonates **33**, easily available from the 2-indolylcarbaldehyde, undergo cyclization through a π-allyl-palladium complex by treatment with [PdCl(π-allyl)]_2_ as the catalyst and Li_2_CO_3_ in CH_2_Cl_2_ in the presence of C1- and C2-symmetrical P/P and P/N ligands to yield 4-vinyl-tetrahydro-β-carbolines **34** (Scheme 18) [75–76]. The best results in terms of enantioselectivity were achieved by using **35** as a ligand, which provided products with (*R*)-configuration of the newly formed stereocenter in enantiomeric excesses up to 97%. Remarkably, the same catalytic system was successfully applied to 3-indolylcarbonates, giving 1-vinyl-tetrahydro-γ-carbolines with high enantiomeric excesses.

**Scheme 19:**
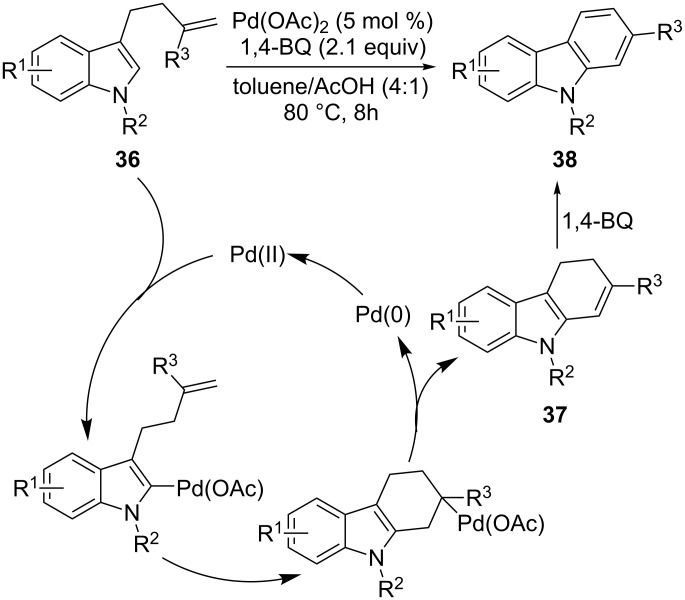
Pd-catalyzed *endo*-cyclization of 3-alkenylindoles for the construction of carbazoles.

The intramolecular reaction of 3-(alken-4-yl)indoles **36** was achieved with Pd(OAc)_2_ as the catalyst and 1,4-benzoquinone as the oxidant, providing carbazole derivatives **38** (Scheme 19) [77]. The products arise from an *endo*-cyclization which gives the initially formed dihydrocarbazoles **37**, which are easily oxidized to the products **38** by the excess of 1,4-benzoquinone. Although better yields were obtained with electron-donating groups, this synthetic approach tolerates a range of substituents on the indole ring.

**Scheme 20:**
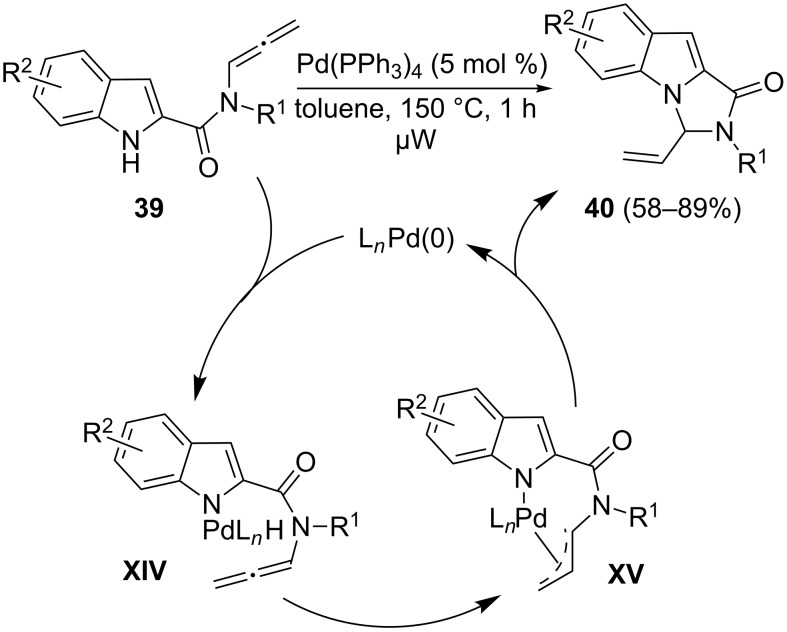
Pd-catalyzed hydroamination of 2-indolyl allenamides.

In 2010, our group disclosed a general route towards 3-vinylimidazo[1,5-*a*]indole derivatives **40** by the unusual and atom-economical intramolecular Pd-catalyzed hydroamination of the allenes **39**, easily accessible by prototropic isomerization of the corresponding propargylamides (Scheme 20) [78]. The selective 5-*exo*-allylic hydroamination occurs in mild conditions in the presence solely of Pd(PPh_3_)_4_ under microwave irradiation by an initial coordination of the Pd(0) catalyst to the indole nitrogen giving the Pd(II)-hydride complex **XIV**. Such an intermediate would be susceptible to insertion of the allene group into the Pd–H bond to generate the π-allyl-Pd(II) complex **XV**, which in turn would undergo the intramolecular formation of the new carbon–nitrogen bond, which regenerates the Pd(0) species.

**Scheme 21:**
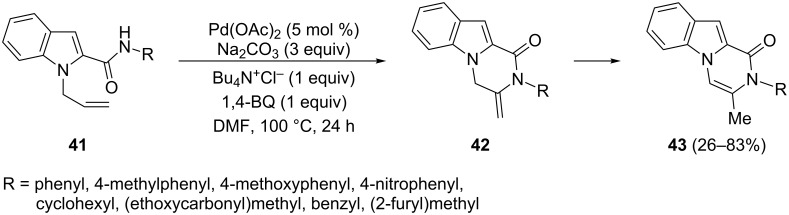
Amidation reaction of 1-allyl-2-indolecarboxamides.

The intramolecular Pd(II)-catalyzed reaction of the 1-allyl-2-indolecarboxamides **41** leads to the pyrazino[1,2-*a*]indoles **43** through the conversion of the olefinic C–H bond into a C–N bond (Scheme 21) [79]. The cyclization process resulted in the initially formed exomethylenic tricyclic derivatives **42**, which undergo an inside double-bond migration to give the final products **43**. This synthetic protocol is founded on two established features: the presence of a base and tetrabutylammonium chloride, essential for the cyclization step, and the stoichiometric amount of an oxidant in order to achieve reoxidation of the Pd(0) species to Pd(II).

### Intramolecular reactions involving arenes

**Scheme 22:**
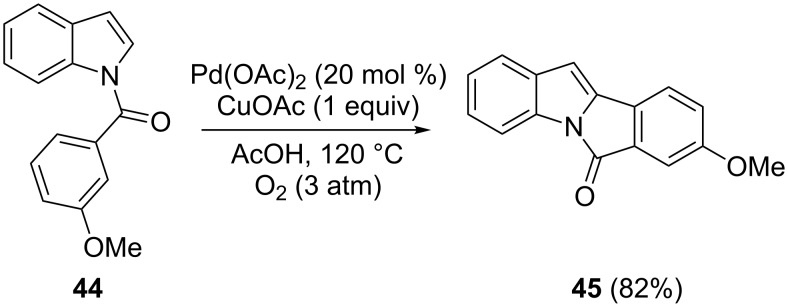
Intramolecular cyclization of *N*-benzoylindole.

Intramolecular arylations by oxidative coupling were investigated by DeBoef and co-workers as a tool for synthesizing heteropolycyclic compounds [80]. The aerobic Pd(II)-catalyzed reaction of the *N*-benzoylindole **44** occurred in the cyclization providing the tetracyclic compound **45** (Scheme 22). The presence of an electron-donating group on the linked arene was proven to be essential for obtaining the product in high yield.

### Alkenylation reactions involving domino processes

**Scheme 23:**
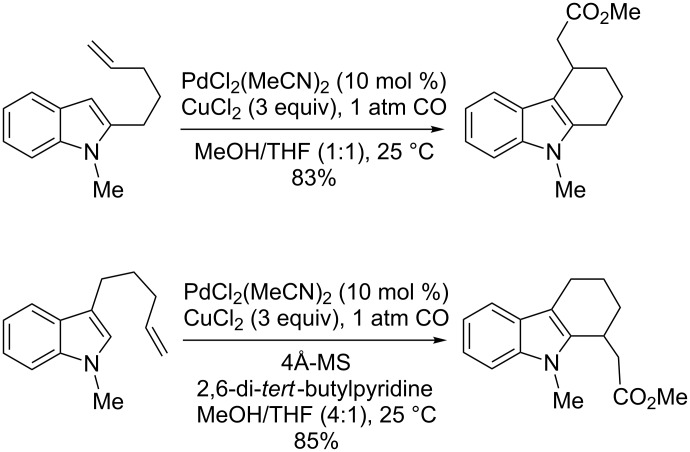
Intramolecular alkenylation/carboxylation of alkenylindoles.

In 2004, Widenhoefer described the cyclization of alkenylindoles by Pd(II) catalysis under carbonylative conditions [81–82]. This approach, based on the use of copper(II) chloride as oxidant, has been applied to 2- and 3-alkenylindoles, resulting in a domino process that involves an alkenylation/carboxylation sequence (Scheme 23). Thus, exploiting the nucleophilicity of the C-2 and C-3 indolyl positions and the subsequent addition of carbon monoxide and the proper alcohol, a broad range of alkoxycarbonyl-substituted indoles fused to various sizes of rings has been achieved under mild conditions.

**Scheme 24:**
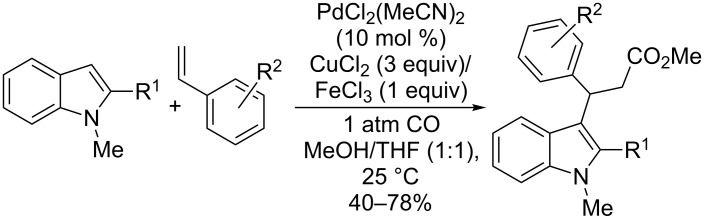
Intermolecular alkenylation/carboxylation of 2-substituted indoles.

A similar intermolecular version of the alkenylation/carboxylation sequence was successfully performed by reaction of styrene compounds with 2-substituted indoles to give 3-benzylindoles bearing an ester group (Scheme 24). It should be pointed out that the presence of a functional group at the C-2 indolyl position is essential to obtain a satisfactory outcome of the reaction. Conversely, different substituents on the styrene substrates affected only the yield of the reaction.

**Scheme 25:**
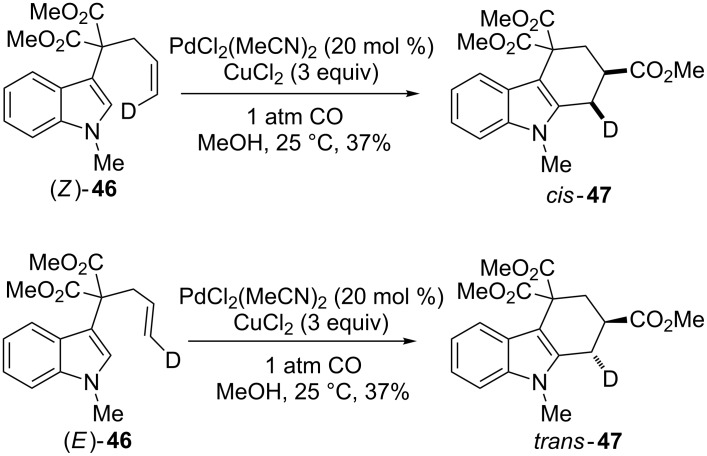
Mechanistic investigation of the cyclization/carboxylation reaction.

The intramolecular reaction has a stereospecific outcome, as demonstrated by the cyclization of the (*Z*) and (*E*)-deutero-indoles **46** (Scheme 25). In fact, (*Z*) and (*E*)-substrates furnished the *cis* and *trans*-products **47**, respectively, as single diastereoisomers. This behavior is the result of an *anti*-addition of the indolyl nucleus and the alkoxycarbonyl group to the ethylenic bond.

**Scheme 26:**
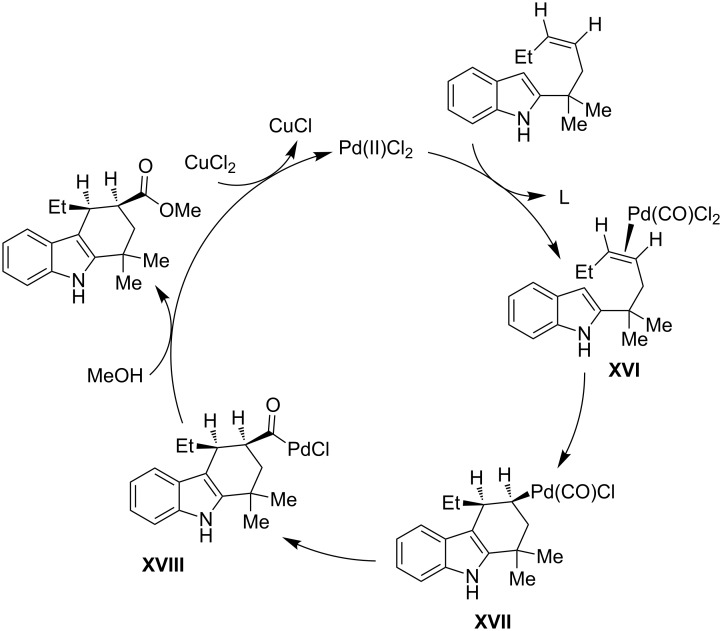
Plausible catalytic cycle for the cyclization/carboxylation of alkenylindoles, adapted from Liu et al. [81].

The stereochemical findings obtained with the cyclization of the (*Z*)-alkenylindoles (as depicted in Scheme 26) give evidence for a mechanism based on the initial coordination of the metal to the olefin with generation of the π-olefin-intermediate **XVI**. The latter is able to undergo an outer-sphere attack by the indole, occurring in the cyclization step with the σ-alkyl-palladium complex **XVII**. The subsequent transfer of carbon monoxide with stereochemical retention determines the generation of the σ-acyl-palladium complex **XVIII**, which in turn is converted in the final *cis*-substituted tetrahydrocarbazole by methanolysis giving the carboxylation step. Again, the released Pd(0) species requires an oxidation by the copper(II) salt to the Pd(II) species, which is then suitable to restart a new catalytic cycle.

**Scheme 27:**
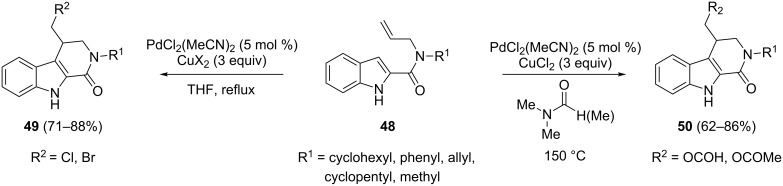
Intramolecular domino reactions of indolylallylamides through alkenylation/halogenation or alkenylation/esterification processes.

Recently, the oxidative Pd(II)-catalyzed strategy for the cyclization of alkenylindoles has been extended to the intramolecular domino reactions of indolylallylamides by using the same couple PdCl_2_(MeCN)_2_/CuX_2_ as catalyst and oxidant, respectively. 2-Indolylallylcarboxamides **48** have been found to be suitable substrates to access variously substituted β-carbolinones **49** and **50** through alkenylation/halogenation or alkenylation/esterification processes selectively obtained by switching reaction solvent and temperature (Scheme 27) [83].

**Scheme 28:**
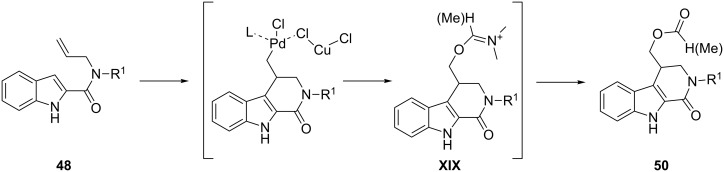
Proposed mechanism for the alkenylation/esterification process through iminium intermediates.

The unforeseen formation of alkenylation/esterification products plausibly arises from a direct intervention of dimethylformamide or dimethylacetamide used as the solvent. The presence of CuCl_2_ slows the β-hydride-elimination process from the σ-alkyl-palladium complexes, favoring a transient palladium oxidation or the generation of hetero-bimetallic palladium/copper intermediates, which may undergo nucleophilic attack by the solvent on the exocyclic carbon to give the iminium intermediates **XIX** (Scheme 28). Finally, the latter may be converted into the esters **50** by hydrolysis.

**Scheme 29:**
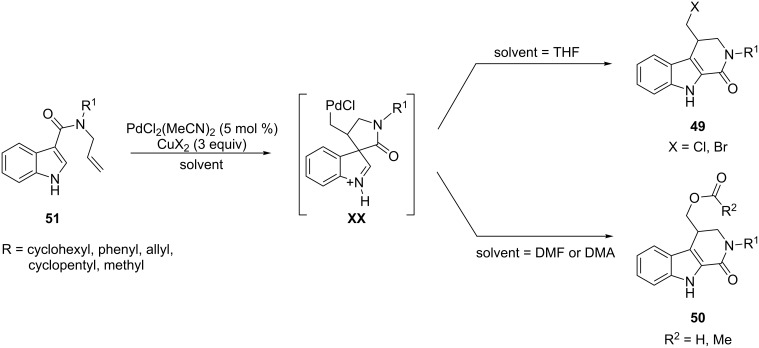
Cyclization of 3-indolylallylcarboxamides involving 1,2-migration of the acyl group from spiro-intermediates.

The same reactivity was satisfactorily tested also on the 3-indolylallylcarboxamides **51**, giving, however, compounds **49** and **50** already obtained from the substrates **48** (Scheme 29). The formation of **49** and **50** may be reasonably justified by the intervention of the spiro-intermediates **XX**, arising from a cyclization involving the C-3 indolyl position, and which evolve by selective transfer of the acyl group from the quaternary center.

**Scheme 30:**
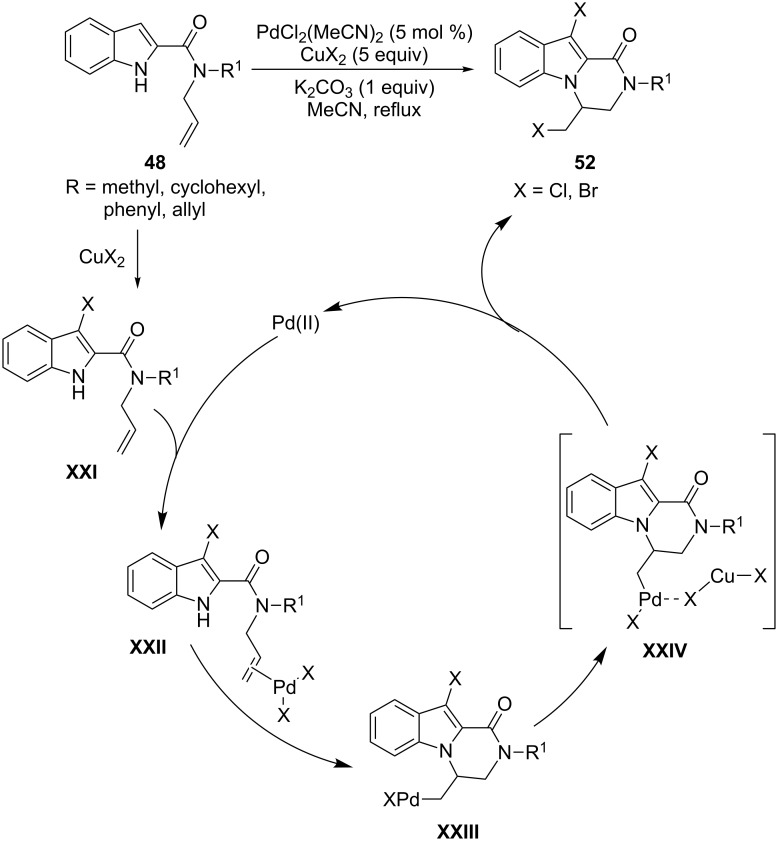
Domino reactions of 2-indolylallylcarboxamides involving N–H functionalization.

The cyclization of 2-indolylallylamides **48**, performed with PdCl_2_(MeCN)_2_ as the catalyst in the presence of CuX_2_ in a large excess and K_2_CO_3_ with acetonitrile as the solvent, allowed the formation of the dihalogenated pyrazino[1,2-*a*]indole derivatives **52** by an unusual aminohalogenation/halogenation sequence (Scheme 30). The formation of the 3-haloderivatives **XXI**, ascribable solely to the action of the Cu(II) salt [84], and the cyclization of the π-olefin complexes **XXII** by aminopalladation leading to the intermediates **XXIII**, are involved as independent steps in the mechanism of the reaction. The final compounds **52** arise from the halide migration on the σ-alkyl-palladium complexes **XXIV**, stabilized by the presence of CuX_2_ in the medium of the reaction [85].

**Scheme 31:**
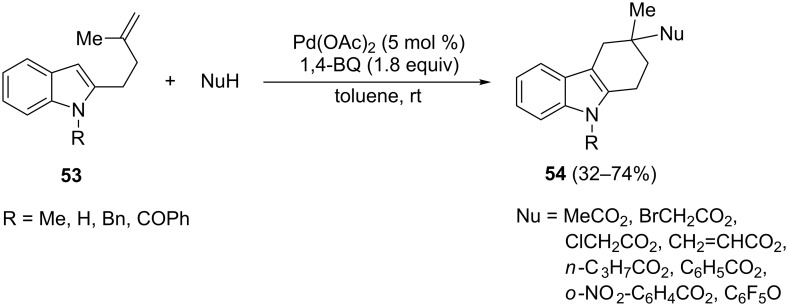
Cyclization/acyloxylation reaction of 3-alkenylindoles.

A mild cyclization of 2-alkenylindoles **53** involving an alkenylation/acyloxylation process resulted in the formation of the 1,2,3,4-tetrahydrocarbazoles **54** bearing oxygen-containing functionalized groups (Scheme 31) [86]. Reactions were carried out by using 1,4-benzoquinone as the oxidizing agent in the presence of different nucleophiles suitable to generate the σ-alkyl-palladium complexes, which give the final products **54** by reductive elimination.

**Scheme 32:**
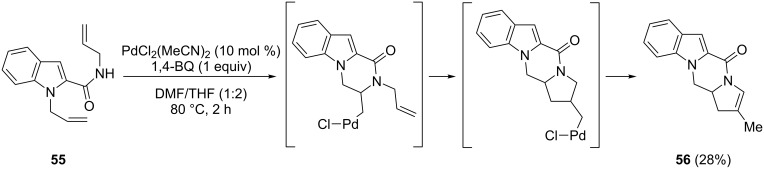
Doubly intramolecular C–H functionalization of a 2-indolylcarboxamide bearing two allylic groups.

The amide of 2-indolecarboxylic acid bearing two allylic groups (**55**) undergoes a domino process with generation of the tetracyclic product **56** (Scheme 32) [79]. Indeed, the reaction carried out with 10 mol % of PdCl_2_(MeCN)_2_ as catalyst and a stoichiometric amount of 1,4-benzoquinone in DMF/THF as solvent underwent an oxidative cascade process involving the sequential intramolecular formation of C–N and C–C bonds, with an oxidative coupling triggered after the initial amidation step.

## Conclusion

Palladium-catalyzed reactions to construct bonds by coupling of C–H/C–H or C–H/N–H bonds have been widely investigated in recent years. This interest arises from the need for unfunctionalized starting materials and from the presence of waste products that are easy to handle, such as hydrogen or water. This strategy, usually tolerant of a wide range of functionalities, has become a very powerful tool in the relevant field of indole chemistry, opening new perspectives for the functionalization of complex molecules avoiding protecting-group chemistry. Despite the results already obtained, many challenges remain, above all related to the improvement in scope and mildness of the reaction conditions for many synthetic protocols described.
